# Emphysema is associated with increased inflammation in lungs of atherosclerosis-prone mice by cigarette smoke: implications in comorbidities of COPD

**DOI:** 10.1186/1476-9255-7-34

**Published:** 2010-07-22

**Authors:** Gnanapragasam Arunachalam, Isaac K Sundar, Jae-woong Hwang, Hongwei Yao, Irfan Rahman

**Affiliations:** 1 Department of Environmental Medicine, Lung Biology and Disease Program, University of Rochester Medical Center, Rochester, NY, USA

## Abstract

**Background:**

Chronic obstructive pulmonary disease is associated with numerous vascular effects including endothelial dysfunction, arterial stiffness and atherogenesis. It is also known that a decline in lung function is associated with increased cardiovascular comorbidity in smokers. The mechanism of this cardiopulmonary dual risk by cigarette smoke (CS) is not known. We studied the molecular mechanisms involved in development of emphysema in atherosclerosis-prone apolipoprotein E-deficient (ApoE^-/-^) mice in response to CS exposure.

**Methods:**

Adult male and female wild-type (WT) mice of genetic background C57BL/6J and ApoE^-/- ^mice were exposed to CS, and lung inflammatory responses, oxidative stress (lipid peroxidation products), mechanical properties as well as airspace enlargement were assessed.

**Results and Discussion:**

The lungs of ApoE^-/- ^mice showed augmented inflammatory response and increased oxidative stress with development of distal airspace enlargement which was accompanied with decline in lung function. Interestingly, the levels and activities of matrix metalloproteinases (MMP-9 and MMP-12) were increased, whereas the level of eNOS was decreased in lungs of CS-exposed ApoE^-/- ^mice as compared to air-exposed ApoE^-/- ^mice or CS-exposed WT mice.

**Conclusion:**

These findings suggest that CS causes premature emphysema and a decline of lung function in mice susceptible to cardiovascular abnormalities via abnormal lung inflammation, increased oxidative stress and alterations in levels of MMPs and eNOS.

## Background

Chronic obstructive pulmonary disease (COPD) is characterized by chronic airflow limitation resulting from excessive airway inflammatory response mediated by cigarette smoke (CS). Comorbidities such as cardiovascular disease, diabetes, lung cancer, and osteoporosis are more prevalent in smokers and patients with COPD [1-3]. Recent studies have shown that smokers with altered forced expiratory volume in one second (FEV_1_) and airflow limitation are associated with arterial stiffness, exaggerated atherosclerosis and vice-versa [2,4,5]. Growing evidence also indicates that inflammation, endothelial dysfunction and oxidative modification of lipids play an important role in the pathogenesis of atherosclerosis and COPD [3,6,7]. In addition to CS, alcohol consumption is also one among the important contributing factors involved in the pathogenesis of COPD and atherosclerosis and their co-morbidities [8,9].

Apolipoprotein E-deficient (ApoE^-/-^) mice develop atherosclerosis due to an accumulation of cholesterol ester-enriched particles in the blood resulting from a lack of triglyceride and cholesterol metabolism/lipid transport [10]. These mice have a shorter life-span and age faster than wild-type counterparts [11]. CS exposure to ApoE^-/- ^mice promotes arterial thrombosis and modulates the size and composition of neointimal lesions/thickening [12], which is associated with increased oxidative stress, reduced glutathione levels and mitochondrial damage leading to atherosclerotic lesion formation [6,13-17]. Massaro and Massaro have recently shown that these mice have an impaired pulmonary morphology and functional phenotype with a rapid decline in lung function as they age [18]. However, the underlying molecular mechanism of the pulmonary phenotype was not studied. We used the ApoE^-/- ^mice, which are prone to develop atherosclerosis [19,20], to understand the molecular mechanism of pulmonary phenotype in response to CS exposure, as well as to study the concept of accelerated decline in lung function and aging in cardiopulmonary comorbid conditions. We determined the inflammatory response, oxidative stress (lipid peroxidation products), levels/activities of matrix metalloproteinases (MMP-9 and MMP-12) and NAD^+^-dependent deacetylase sirtuin 1 (SIRT1) which is shown to regulate endothelial nitric oxide synthase (eNOS) activity (endothelial function) in lungs of ApoE^-/- ^mice exposed to CS.

## Methods

### Reagents

Unless otherwise stated, all biochemical reagents used in this study were purchased from Sigma Chemicals Co., St. Louis, MO, USA. Antibodies used to detect proteins include mouse specific SIRT1 and eNOS (Cell Signaling, Danvers, MA), MMP-9 and MMP-12 (Santa Cruz Biotechnology, Santa Cruz, CA) for western blotting and immunoprecipitation.

### Animals

Adult male and female wild-type (WT) mice of genetic background C57BL/6J and ApoE^-/- ^mice [19,20] (Strain number, B6.129P2-Apoe^tm1Unc^/J; stock number, 002052, backcrossed to C57BL/6J for 10 generations, Jackson Laboratory, Bar Harbor, ME) were housed in the inhalation facility of the University of Rochester. These mice were fed with regular standard Chow diet during housing and experimental procedures. ApoE^-/- ^mice showed obvious signs of atherosclerotic lesions in the aortic sinus and ascending aorta after feeding with Chow diet at 24 weeks of age with an early onset of signs seen after approximately 3-4 months of age (Jackson Lab). ApoE^-/- ^mice develop atherosclerotic plaques at 2-3 months after feeding with a high-fat Western-type diet [20]. All experimental protocols described in this study were approved by the animal research committee of the University of Rochester.

### CS exposure

Adult mice (12 weeks old, body weight ranging from 30-40 g, male and female) were exposed to CS for 3 days using Baumgartner-Jaeger CSM2082i automated cigarette smoking machine (CH Technologies, Westwood, NJ) [21,22]. The smoke was generated from 3R4F research cigarettes (University of Kentucky, Lexington, KY). Mainstream CS was diluted with filtered air, and directed into the exposure chamber. Monitoring of the CS exposure (TPM per cubic meter of air, mg/m^3^) was performed in real-time using MicroDust Pro-aerosol monitor (Casella CEL, Bedford, UK) and verified daily by gravimetric sampling. The smoke concentration was set at a nominal value of approximately 300 mg/m^3 ^TPM by adjusting the flow rate of the dilution air [21,22]. The control mice were exposed to filtered air in an identical manner.

### Bronchoalveolar lavage and tissue harvest

The mice were intraperitoneally injected with 100 mg/kg body weight of pentobarbiturate (Abbott laboratories, Abbott Park, IL) and killed by exsanguination. The lungs were lavaged three times with 0.6 ml of 0.9% sodium chloride and removed *en bloc*. The bronchoalveolar lavage (BAL) fluid cell pellet was resuspended in saline, and the total cell number was counted with a hemocytometer. Differential cell count (500 cells/slide) was performed on cytospin-prepared slides (Thermo Shandon, Pittsburgh, PA) stained with Diff-Quik (Dade Bering, Newark, DE).

### Cytokine analysis

The levels of proinflammatory mediators such as monocyte chemoattractant protein-1 (MCP-1) and chemokine keratinocyte chemoattractant (KC) in lung homogenates were measured by ELISA using respective duo-antibody kits (R&D Systems, Minneapolis, MN) according to the manufacturer's instructions.

### Immunohistochemical staining for tissue macrophages

Immunohistochemical staining for macrophages in lung sections was performed as described previously [21,22]. The number of Mac-3-positive cells in each lung section (5 random microscopic fields per lung section in 3 different sections) was counted manually at ×200 magnification and averaged.

### Lipid peroxidation products assay in lung homogenate

The right lung lobe was homogenized with ice-cold 20 mM Tris-HCl (pH 7.4) and centrifuged at 3,000 g at 4°C for 10 min, and the supernatants were collected. Butylated hydroxytoluene (5 mM) was added to the supernatant to prevent further peroxidation, and the samples were immediately frozen in liquid nitrogen. Lipid peroxidation products [malondialdehyde (MDA) and 4-hydroxy-2-nonenal (4-HNE)] were measured using a lipid peroxidation kit (Enzo Life Sciences, PA) according to the manufacturer's instructions [22].

### Measurement of lung mechanical properties

Lung mechanical properties were determined using Scireq Flexivent apparatus (Scireq, Monteral, Canada). The dynamic lung compliance and lung resistance were measured in mice anesthetized by sodium pentobarbital (50 mg/kg, intraperitoneally) and paralyzed with pancuronium (0.5 mg/kg, intraperitoneally). A tracheotomy was performed and an 18-guage cannula was inserted 3 mm into an anterior nick in the exposed trachea and connected to a computer controlled rodent ventilator. Initially, the mice were ventilated with room air (150 breaths/min) at a volume of 10 ml/kg body mass. After 3 minutes of ventilation, measurement of lung mechanical properties were initiated by the computer generated program to measure dynamic lung compliance and resistance. These measurements were repeated three times for each animal.

### Hematoxylin and Eosin (H&E) staining and mean linear intercept analysis

Mouse lungs (which had not been lavaged) after CS exposure were inflated by 1% low-melting agarose at a pressure of 25 cm H_2_O, and then fixed with neutral buffered formalin. Tissues were embedded in paraffin, sectioned (4 μm), and stained with hematoxylin and eosin (H&E). The alveolar size was estimated from the mean linear intercept (Lm) of the airspace which is a measure of airspace enlargement/emphysema. Lm was calculated for each sample based on 10 random fields observed at a magnification of ×200 using cross-lines as described previously [21,22].

### Immunoblotting

Proteins (20 μg) from lung tissue homogenates were used for immunoblotting as described previously [21-24]. In brief, protein was electrophoresed on 7.5% SDS-PAGE gel and transblotted on nitrocellulose membrane (Amersham Biosciences, Piscataway, NJ). Membranes were blocked with 5% (w/v) non-fat milk in PBS containing 0.1% (v/v) Tween 20 and then incubated with anti-SIRT1, anti-eNOS, anti-MMP-9 or anti-MMP-12 antibodies. After washing, bound antibody was detected using anti-rabbit/anti-mouse antibody linked to horseradish peroxidase and bound complexes were detected using enhanced chemiluminescence (Perkin Elmer, Waltham, MA). Protein levels were measured by BCA kit as per the manufacturer's instructions using BSA as standards (Thermo Scientific, Rockford, IL).

### SIRT1 deacetylase activity assay

SIRT1 activity was assayed using a deacetylase colorimetric activity assay kit according to the manufacturer's instructions (Biomol International, Plymouth Meeting, PA). Briefly, SIRT1 was immunoprecipitated from whole lung homogenates (100 μg protein). After the final washing, *Color de Lys *substrate reagent and NAD^+ ^were added to the SIRT1 conjugated beads and incubated at 37°C for 80 min. The substrate-SIRT1 mixture was then placed on a 96-well plate, and the *Color de Lys *developer reagent was added to the wells at 37°C for 20 min. The plate was then read at 405 nm using a spectrophotometer (Model 680 microplate reader, Bio-Rad, Hercules, CA).

### MMPs activity assay by zymography

The zymography was performed to determine the activity of MMPs in mouse lung as described previously [25]. Briefly, lung tissues were homogenized in 400 μl lysis buffer (50 mM Tris-HCl, pH 7.4, with protease inhibitors) on ice. One hundred micrograms of protein was then mixed with equal volume sample buffer (80 mM Tris-HCl, pH 6.8, 4% SDS, 10% glycerol, 0.01% bromophenol blue) and then loaded on a 7.5% SDS-polyacrylamide gel containing 1 mg/ml gelatin which was overlaid with 5% stacking gel. After electrophoresis, gels were rinsed in distilled water, washed three times for 15 minutes each in 150 ml 2.5% Triton X-100 solution. Gels were then incubated in 100-150 ml of 50 mM Tris-HCl (pH 7.5), 10 mM CaCl_2_, 1 μM ZnCl_2_, 1% Triton X-100 and 0.02% NaN_3_. After incubation, gels were stained with 100 ml Coomassie blue R-250 for 3 h and then destained 1 h with destaining solution (50% methanol, 10% acetic acid). Gels were washed in distilled water for 20 minutes and then scanned. The intensity of bands was quantified using image J software (Version 1.41, National Institutes of Health, Bethesda, MD, USA).

### Statistical analysis

Data were presented as means ± SEM. Statistical analysis of significance was calculated using one-way analysis of variance followed by *post hoc *test for multigroup comparisons using Stat View software. *P *< 0.05 was considered as significant.

## Results

### ApoE^-/- ^mice are susceptible to increased lung inflammatory cell influx in response to CS

**Figure 1:**
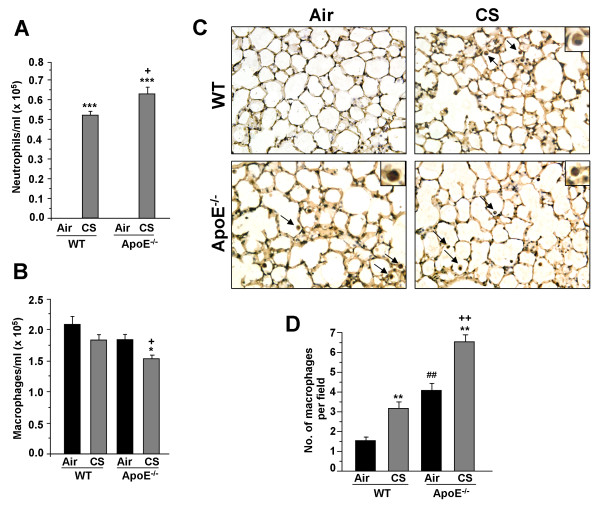
**Neutrophil and macrophage influx into BAL fluid and lungs of ApoE**^**-/- **^**mice exposed to CS**. Neutrophil and macrophage influx were analyzed in BAL fluid by Diff-Quik staining on cytospin slides (A and B respectively). Data are shown as mean ± SEM (n = 3-4 mice per group). *P < 0.05, ***P < 0.001, significant compared with corresponding air-exposed mice. ^**+**^*P *< 0.05, significant compared with CS-exposed WT mice. Lung sections of air- and CS-exposed WT and ApoE^-/- ^mice were stained with anti-mouse Mac-3 antibody (C). Mac-3-positive cells (dark brown) were identified by immunohistochemical staining (indicated by arrows and insets), Original magnification: ×200. Histogram (D) represents mean ± SEM. **P < 0.01, significant compared with corresponding air-exposed mice. ^**++**^*P *< 0.01, significant compared with CS-exposed WT mice. ^##^*P *< 0.01, significant compared with air-exposed WT mice (n = 4).

Augmented inflammatory response in the lung from environmental stress or toxicants results in the activation of inflammatory cascades in microvasculature and vessel walls leading to a potentiation of atherogenesis [12,13,26,27]_. _Atherogenic prone ApoE^-/- ^were exposed to CS for 3 days, and the number of neutrophils and macrophages in BAL fluid as well as in the lungs were determined. CS exposure led to a higher number of neutrophil influx in BAL fluid of ApoE^-/- ^mice as compared to WT mice (Fig. 1A). However, CS exposure significantly decreased the number of macrophages in BAL fluid of ApoE^-/- ^mice, but not in WT mice (Fig. 1B). Interestingly, the macrophage infiltration in lung interstitium of air-exposed ApoE^-/- ^mice was significantly increased as compared to air- and CS-exposed WT mice. This was augmented in CS-exposed ApoE^-/- ^mice (Fig. 1C, D).

### CS exposure augments the proinflammatory cytokine levels in lungs of ApoE^-/- ^mice

**Figure 2:**
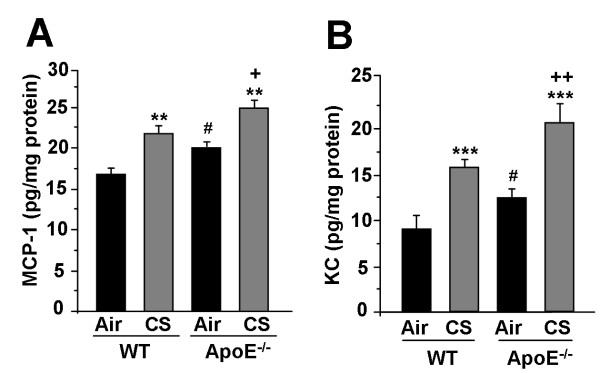
**Levels of pro-inflammatory mediators in lungs of ApoE**^**-/- **^**mice exposed to CS**. The levels of pro-inflammatory mediators such as MCP-1 (A) and KC (B) were measured by ELISA in lung homogenates of air- and CS-exposed WT and ApoE^-/- ^mice. Data are shown as mean ± SEM (n = 3-4 mice per group). **P < 0.01, ***P < 0.001, significant compared with corresponding air-exposed mice. ^**+**^*P *< 0.05, ^**++**^*P *< 0.01 significant compared with CS-exposed WT mice. ^#^*P *< 0.05, significant compared with air-exposed WT mice

In order to confirm whether the inflammatory cell influx was associated with proinflammatory cytokine release in ApoE^-/- ^mice, the levels of proinflammatory mediators, such as MCP-1 and KC, which can recruit macrophages and neutrophils in the lung, were measured in lung homogenates of air- and CS-exposed WT and ApoE^-/- ^mice. CS-exposure to ApoE^-/- ^mice significantly increased the levels of MCP-1 and KC as compared to CS-exposed WT mice (Fig. 2A, B). These results suggest that increased levels of MCP-1 and KC may contribute to enhanced macrophage and neutrophil influx in the lungs of ApoE^-/- ^mice after CS exposure.

### ApoE^-/- ^mice lung shows increased oxidative stress as lipid peroxidation products (4-HNE and MDA) in response to CS

**Figure 3:**
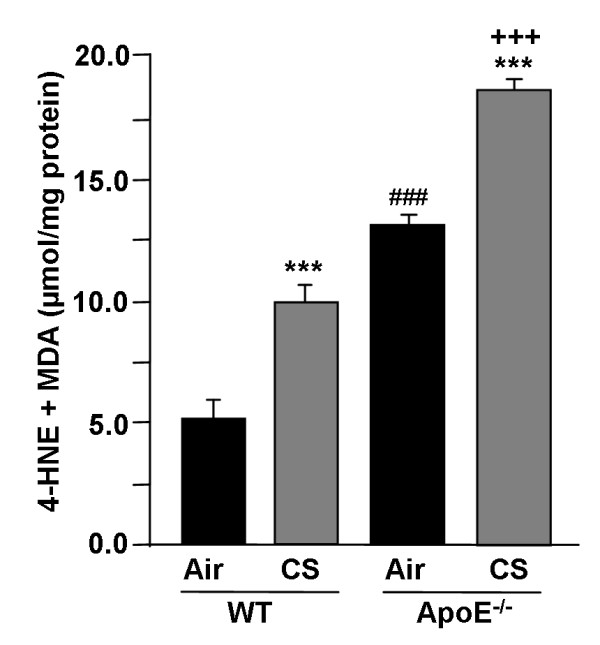
**Levels of lipid peroxidation products (4-HNE and MDA) in lungs of ApoE**^**-/- **^**mice exposed to CS**. Levels of 4-HNE and MDA were measured spectrophotometrically in lung homogenates of WT and ApoE^-/- ^mice exposed to CS. Histograms represent mean ± SEM of n = 3-4 per group. ***P < 0.001, significant compared with corresponding air-exposed mice. ^**+++**^*P *< 0.001, significant compared with CS-exposed WT mice. ^###^*P *< 0.001, significant compared with air-exposed WT mice.

We previously showed that CS-induced oxidative stress is involved in the development of emphysema and vascular endothelial dysfunction [21,22,28]. Therefore, we assessed the lung levels of lipid peroxidation products (4-HNE and MDA) as a measure of increased oxidative stress in WT and ApoE^-/- ^mice exposed to CS. A significant increase in 4-HNE and MDA levels were observed in CS-exposed ApoE^-/- ^mice lung compared to WT (Fig. 3). This result suggests that CS-induced oxidative stress and lipid peroxidation might be the causative factor for an increased inflammatory response, which would lead to the development of premature emphysema and vascular abnormalities in these mice.

### ApoE^-/- ^mice show increased airspace enlargement and alterations in lung mechanical properties in response to CS exposure

**Figure 4:**
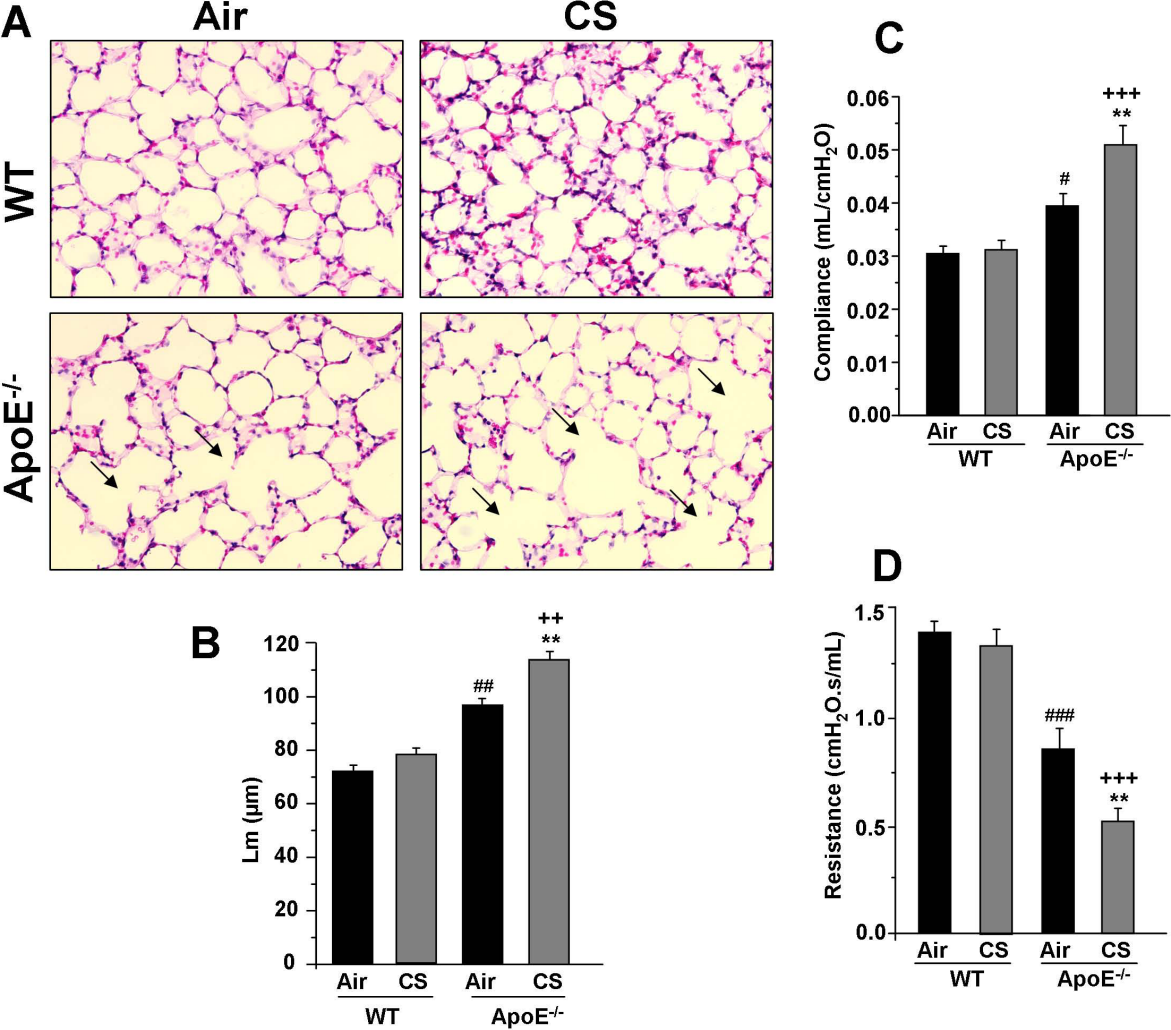
**Airspace enlargement and lung mechanical properties in ApoE**^**-/- **^**mice exposed to CS**. Representative figure of H&E stained lung sections from air- and CS-exposed WT and ApoE^-/- ^mice (A). Arrows indicate alveolar enlargement. Mean linear intercept (Lm) was calculated in H&E stained lung sections. Original magnification: ×200. Histogram represents (B) mean ± SEM (n = 3-4 mice per group). **P < 0.01, significant compared with corresponding air-exposed mice. ^**++**^*P *< 0.01, significant compared with CS-exposed WT mice. ^##^*P *< 0.01, significant compared with air-exposed WT mice. Lung compliance (C) and resistance (D) were measured in air- and CS-exposed WT and ApoE^-/- ^mice using Flexivent. Data are shown as mean ± SEM (n = 3-4 mice per group). **P < 0.01, significant compared with corresponding air-exposed mice. ^**+++**^*P *< 0.001, significant compared with CS-exposed WT mice. ^#^*P *< 0.05, ^###^*P *< 0.001, significant compared with air-exposed WT mice.

We measured the airspace enlargement and decline in lung function, which are the characteristics of pulmonary emphysema/COPD, in WT and ApoE^-/- ^mice exposed to air or CS. ApoE^-/- ^mice exposed to CS showed a significant increase in alveolar size as compared to air- and CS-exposed WT mice (Fig. 4A, B). There was also a spontaneous airspace enlargement seen in ApoE^-/- ^mice. The lung compliance (measured as lung function) was significantly increased in air- and CS-exposed ApoE^-/- ^mice compared to air- and CS-exposed WT mice (Fig. 4C, D). The lung resistance was significantly lowered in air- and CS-exposed ApoE^-/- ^mice compared to air- and CS-exposed WT mice. These data suggest that lungs of ApoE^-/- ^mice have impaired alveologenesis and alveolar destruction with altered lung mechanical properties, which were augmented by acute CS exposure.

### ApoE^-/- ^mice show increased levels and activities of matrix metalloproteinases, and reduction of SIRT1 levels and activity as well as eNOS levels in lungs by CS

**Figure 5:**
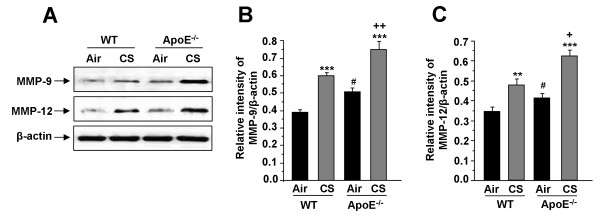
**Levels of MMPs in lungs of ApoE**^**-/- **^**mice exposed to CS**. The levels of MMP-9 and MMP-12 were determined in lungs of air- and CS-exposed WT and ApoE^-/- ^mice by immunoblotting (A). Histograms (B and C) represent mean ± SEM (n = 3-4 per group). **P < 0.01, ***P < 0.001, significant compared with corresponding air-exposed mice. ^**+**^*P *< 0.05, ^**++**^*P *< 0.01, significant compared with CS-exposed WT mice. ^#^*P *< 0.05, significant compared with air-exposed WT mice.

MMPs, particularly increased levels of MMP-9 and MMP-12, are involved in CS-mediated airspace enlargement/alveolar wall destruction (emphysema). Hence, we determined whether the levels and activities of MMP-9 and MMP-12 were altered in ApoE^-/- ^mice after CS exposure. The levels of MMP-9 and MMP-12 were significantly increased in lungs of CS-exposed ApoE^-/- ^mice compared to that of WT mice (Fig. 5A-C). Similarly, there was a 1.8 and 2.2-fold increase in MMP-9 and MMP-12 activities respectively in lungs of WT mice exposed to CS as compared to air-exposed WT mice. Air-exposed ApoE^-/-^mice showed a 1.6 and 1.8-fold increase in corresponding MMP-9 and MMP-12 activities in the lungs as compared to air-exposed WT mice, which was further augmented in CS-exposed ApoE^-/- ^mice (2.8-fold increase in MMP-9 activity and 2.6-fold increase in MMP-12 activity).

**Figure 6:**
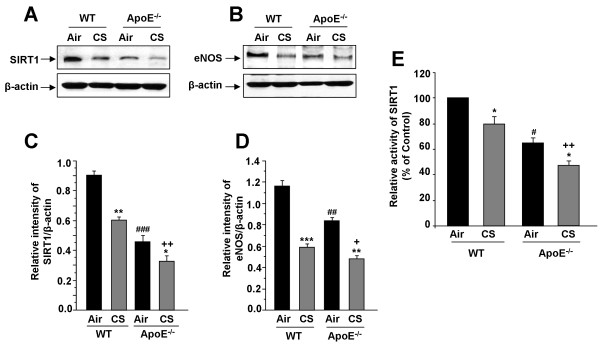
**SIRT1 levels and activity, and eNOS level in lungs of ApoE**^**-/- **^**mice exposed to CS**. SIRT1 and eNOS levels were measured in lungs of WT and ApoE^-/- ^mice exposed to CS (A and B). Histograms (C and D) represent mean ± SEM of relative levels of SIRT1 and eNOS respectively (n = 3-4 per group). SIRT1 deacetylase activity was measured in lungs of WT and ApoE^-/- ^mice exposed to CS (E). *P < 0.05, **P < 0.01, ***P < 0.001 significant compared with respective air-exposed mice. ^**+**^*P *< 0.05, ^**++**^*P *< 0.01, significant compared with CS-exposed WT mice. ^#^*P *< 0.05, ^##^*P *< 0.01, ^###^*P *< 0.001, significant compared with air-exposed WT mice.

We determined the levels of SIRT1 and eNOS in lungs of ApoE^-/- ^mice exposed to CS. The basal endogenous abundances of SIRT1 and eNOS were significantly decreased in ApoE^-/- ^mice compared with WT mice (Fig. 6A-D). ApoE^-/- ^mice exposed to CS showed further reduction in SIRT1 level and activity (Fig. 6E) and eNOS levels (Fig. 6B, D) compared to air- and CS-exposed WT mice. Hence, CS-mediated reduction in SIRT1 and eNOS levels was associated with pulmonary functional and morphological phenotype alterations in ApoE^-/- ^mice.

## Discussion

Prolonged exposure to CS leads to the development of COPD associated with arterial stiffness, endothelial dysfunction and atherosclerosis-mediated cardiovascular diseases [1-5]. The lungs of ApoE^-/- ^mice also have impaired alveologenesis with altered lung mechanical properties [18]. However, the underlying molecular mechanism of this pulmonary phenotype in ApoE^-/- ^by CS is not known. We used ApoE^-/- ^mice to study the pulmonary phenotype in response to CS. We found that the air-exposed WT and ApoE^-/- ^mice showed no change in neutrophil influx, whereas CS-exposed ApoE^-/- ^mice had an increased neutrophil influx in BAL fluid compared to CS-exposed WT mice. The macrophage influx in lung interstitium was also significantly increased in lungs of CS-exposed ApoE^-/- ^mice compared to CS-exposed WT or control ApoE^-/- ^mice. MCP-1 and KC (pro-inflammatory cytokines) are capable of recruiting macrophages and neutrophils respectively into the lungs in the presence and absence of inflammatory stimuli [29,30]. The susceptibility of ApoE^-/- ^mice to CS-mediated increased inflammation was further confirmed by the increased levels of proinflammatory cytokine (MCP-1 and KC) release in lungs of adult 12 weeks old ApoE^-/- ^mice exposed to CS for acute period (3 days) when fed the regular/standard Chow-diet. Interestingly, air-exposed ApoE^-/- ^mice also showed increased pro-inflammatory cytokine release possibly due to infiltrated macrophages in the lung, which was further increased in response to CS exposure. Previously, it has been shown that lungs of ApoE^-/- ^mice had increased levels of pro-inflammatory cytokine (TNF-α, IL-1) and expression of adhesion molecules, such as inter-cellular adhesion molecule-1 (ICAM-1) and vascular cell adhesion molecule-1 (VCAM-1) [31]. These findings suggest that ApoE^-/- ^mice are prone to develop atherosclerotic lesions by activation of proatherogenic molecules which are associated with augmented lung inflammatory response. However, it is not known whether T and B cells are involved in the inflammatory response seen in ApoE^-/- ^mice, since these cells also play an important role in the development of emphysema/COPD in humans. Further studies are required to confirm this possibility.

CS either directly or indirectly induces the production of ROS such as superoxide anions, hydroxyl radicals and hydrogen peroxide. We have previously shown that the imbalance between oxidants and antioxidants are associated with lung inflammatory response and development of emphysema [21,22]. In the present study, CS exposure resulted in increased levels of lipid peroxidation products, as shown by the generation of 4-HNE and MDA in lungs of ApoE^-/- ^mice. It is possible that CS augments the generation of lipid peroxidation derived 4-HNE which would activate inflammatory signaling pathways in the lungs of ApoE^-/- ^mice, thereby leading to an increased inflammatory response and development of premature emphysema in these mice.

Reduced FEV_1 _with airflow limitation is often associated with atherosclerosis and other cardiovascular morbidities [2-4]. Our data show increased airspace enlargement/alveolar destruction with altered lung compliance and resistance in air-exposed ApoE^-/- ^mice, which were further aggravated in response to acute CS exposure. Since increased levels of MMPs, such as MMP-9 and MMP-12, are potentially involved in alveolar destruction associated with altered lung function, we measured the levels and activities of MMPs in lungs of ApoE^-/- ^mice. ApoE^-/- ^mice exposed to CS showed the increased levels of MMP-9 and MMP-12 in the lung compared to WT or control ApoE^-/- ^mice. Furthermore, the activities of MMP-9 and MMP-12 were increased in lungs of CS-exposed ApoE^-/- ^mice as compared to that of WT mice. It is possible that increased macrophage infiltration into the lungs in response to CS exposure may lead to elevated MMPs which might be the cause for airspace enlargement and lower lung function observed in these mice. It is noteworthy to mention here that ApoE^-/- ^mice when fed with high cholesterol diet show increased inflammatory cell recruitment with enhanced MMP-9 activity [31,32]. Furthermore, overexpression of MMP-9 in ApoE^-/- ^mice resulted in an increased smooth muscle cell infiltration (lesion maturation) and increased plaque formation in mouse aorta [32]. These findings suggest that CS-mediated induction of MMPs not only leads to increased alveolar destruction, but is also associated with the atherosclerotic plaque formation in these mice as evidenced earlier [12,13].

It has been shown that eNOS regulates endothelial function and several components of the atherogenic process, such as vascular smooth muscle cell contraction, proliferation, platelet aggregation, and monocyte adhesion [27,33-35]. Previously, it has been shown that ApoE^-/- ^mice have a deficiency of eNOS which is exhibited with high levels of atherosclerotic lesion formation [36,37]. In the present study, the eNOS level was measured in order to understand whether CS-mediated emphysema in ApoE^-/- ^mice was associated with endothelial dysfunction. The basal abundance of eNOS was significantly decreased in the lungs of ApoE^-/- ^mice compared to WT mice with further reduction in ApoE^-/- ^mice exposed to CS. These data are supported by a previous study demonstrating the decreased eNOS level in ApoE^-/- ^mice exposed to ozone was associated with increased vascular dysfunction, oxidative stress, mitochondrial damage, and atherogenesis [38]. Furthermore, knockdown of eNOS in ApoE^-/- ^mice showed increased lesions area with peripheral coronary atherosclerosis with myocardial fibrosis compared with ApoE^-/- ^alone [37]. These observations implicate that a reduction of eNOS leads to altered endothelial function in lung microvasculature and/or vascular disruption as well as atherogenesis.

Recently, we and others have shown that eNOS is regulated by acetylation/deacetylation via SIRT1 deacetylase or calorie restriction [34,39,40]. Previous studies have shown that CS causes reduction in SIRT1 levels/activity by posttranslational modification such as alkylation/carbonylation, which was associated with increased proinflammatory gene expression [23,24,41]. Furthermore, calorie/dietary restriction or overexpression of SIRT1 in ApoE^-/- ^mice exhibited an anti-atherosclerosis effect by inhibiting oxidized low-density lipoprotein (LDL)-induced apoptosis, upregulation of eNOS expression and improved endothelium-dependent vasorelaxation [42,43]. Interestingly, the SIRT1 level and activity were significantly decreased in the lungs of ApoE^-/- ^mice with further reduction in response to CS. Decreased SIRT1 levels and activity may lead to increased acetylation and inactivation of eNOS in the lungs of ApoE^-/- ^mice exposed to CS culminating endothelial dysfunction. However, further studies are required to study how post-translational modifications (e.g. phospho-acetylation) affect its activity in response to CS exposure. This may be one of the reasons that ApoE^-/- ^mice show signs of early aging [11] as SIRT1 is an anti-aging protein [24]. Hence, SIRT1 activation (and NAD^+ ^replenishment) may not only activate eNOS but will also inhibit endothelial cell senescence, atherosclerosis and inflammatory response in the lung [23,44,45]. This is further validated by SIRT1 activation or calorie restriction in ApoE^-/- ^mice leads to protection against atherosclerosis progression by upregulating eNOS [43-45]. Interestingly, our preliminary data showed that overexpression of SIRT1 in ApoE^-/- ^mice in double transgenic mice protected, whereas knockdown of SIRT1 in ApoE^-/- ^mice aggravated the lung phenotype (inflammation and emphysema).

In summary, our study shows the augmented inflammatory response, increased oxidative stress, and airspace enlargement with altered mechanical properties in lungs of ApoE^-/- ^mice in response to CS, which was associated with increased MMPs, reduced SIRT1 activity and eNOS levels. These mice have an accumulation of excess lipids laden in blood and pulmonary arteries/lung microvasculature which can undergo rapid oxidation by CS-derived free radicals and oxidants leading to the generation of secondary oxidized lipid mediators/peroxidation products/signaling molecules both systemically and locally. This will trigger alterations in SIRT1, eNOS and abnormal inflammatory responses leading to pulmonary functional and morphological phenotype. This may be one of the mechanisms linking CS-mediated accelerated decline in lung function and aging in comorbidities of cardiopulmonary diseases [46].

## Abbreviations

ApoE: apolipoprotein E; COPD: chronic obstructive pulmonary diseases; CS: cigarette smoke; eNOS: endothelial nitric oxide synthase; 4-HNE: 4-hydroxy-2-nonenal; MDA: malondialdehyde; MMPs: matrix metalloproteinases.

## Competing interests

The authors declare that they have no competing interests.

## Authors' contributions

GA contributed in the study design and planning, and performed the experiments. JH, IKS and HY participated and coordinated in completing the study. GA wrote the first draft of the manuscript. IR supervised the study and contributed in data discussions and correcting the drafts. Furthermore, IR conceived the study, contributed in the study design, planning and revised the manuscript. All authors read and approved the final manuscript.
